# Exposure to concentrated ambient PM_2.5_ alters the composition of gut microbiota in a murine model

**DOI:** 10.1186/s12989-018-0252-6

**Published:** 2018-04-17

**Authors:** Wanjun Wang, Ji Zhou, Minjie Chen, Xingke Huang, Xiaoyun Xie, Weihua Li, Qi Cao, Haidong Kan, Yanyi Xu, Zhekang Ying

**Affiliations:** 1 Department of Environmental Health, School of Public Health, Fudan University, 130 Dong’an Rd, Shanghai, 200032 China; 2 Shanghai Key Laboratory of Meteorology and Health, Shanghai Meteorological Service, Shanghai, China; 3 Department of Medicine Cardiology Division, School of Medicine, University of Maryland, 20 Penn St. HSFII S022, Baltimore, MD 21201 USA; 4 Department of Interventional & Vascular Surgery, Shanghai Tenth People’s Hospital, Tongji University School of Medicine, Shanghai, China; 5 Reproductive and Developmental Research Institute of Fudan University, Shanghai, China; 6 Department of Diagnostic Radiology and Nuclear Medicine, University of Maryland School of Medicine, Baltimore, MD USA

**Keywords:** PM_2.5_, Gut microbiota, Glucose homeostatic, Diabetes

## Abstract

**Background:**

Exposure to ambient fine particulate matter (PM_2.5_) correlates with abnormal glucose homeostasis, but the underlying biological mechanism has not been fully understood. The gut microbiota is an emerging crucial player in the homeostatic regulation of glucose metabolism. Few studies have investigated its role in the PM_2.5_ exposure-induced abnormalities in glucose homeostasis.

**Methods:**

C57Bl/6J mice were exposed to filtered air (FA) or concentrated ambient PM_2.5_ (CAP) for 12 months using a versatile aerosol concentration enrichment system (VACES) that was modified for long-term whole-body exposures. Their glucose homeostasis and gut microbiota were examined and analysed by correlation and mediation analysis.

**Results:**

Intraperitoneal glucose tolerance test (IPGTT) and insulin tolerance test (ITT) showed that CAP exposure markedly impaired their glucose and insulin tolerance. Faecal microbiota analysis demonstrated that the impairment in glucose homeostasis was coincided with decreased faecal bacterial ACE and Chao-1 estimators (the indexes of community richness), while there was no significant change in all faecal fungal alpha diversity estimators. The Pearson’s correlation analyses showed that the bacterial richness estimators were correlated with glucose and insulin tolerance, and the mediation analyses displayed a significant mediation of CAP exposure-induced glucose intolerance by the alteration in the bacterial Chao-1 estimator. LEfSe analyses revealed 24 bacterial and 21 fungal taxa differential between CAP- and FA-exposed animals. Of these, 14 and 20 bacterial taxa were correlated with IPGTT AUC and ITT AUC, respectively, and 5 fungal taxa were correlated with abnormalities in glucose metabolism.

**Conclusions:**

Chronic exposure to PM_2.5_ causes gut dysbiosis and may subsequently contribute to the development of abnormalities in glucose metabolism.

## Background

Diabetes is one of the leading causes of death globally [1]. In addition to genetic risk variants and behavioural/environmental factors, the gut microbiota is emerging as an important contributor to the pathogenesis of abnormal glucose homeostasis. The interest in the role of gut microbiota in the host’s metabolic homeostasis has been sparked by the observation that germ-free mice have reduced adiposity and improved tolerance to glucose and insulin [2], and are protected from diet-induced obesity and abnormal glucose homeostasis when fed a Western-style diet [3, 4]. The following focused studies have then indicated that the gut microbiota is crucial in the pathogenesis of human diabetes [5]. The biological mechanisms for the role of the gut microbiota in diabetes has also been investigated. The gut microbiota has been shown to influence the host’s capacity of energy harvest and thus impact the development of obesity that is the leading risk factor for diabetes [5]. Furthermore, additional studies have demonstrated that changes in the composition and function of gut microbiota are associated with abnormal glucose homeostasis, independent of other contributing factors such as body weight [6]. For example, type 2 diabetes (T2D) is associated with decreased gut butyrate-producing bacteria and/or increased gut opportunistic pathogens [7], and increased lipopolysaccharide and/or branched-chain amino acid (BCAA) biosynthesis potential of gut microbiota at least partly accounts for the insulin resistance in apparently healthy individuals [8]. Moreover, the high fiber diet-associated amelioration in insulin resistance and hyperglycaemia is correlated with its probiotic function, and dietary supplementation with probiotics or symbiotic improves insulin resistance in diabetic patients [6]. These studies collectively indicate that the gut microbiota plays a crucial role in the pathogenesis of diabetes.

Fine particulate matter (PM_2.5_) pollution is one of the leading environmental factors affecting global public health. Numerous epidemiological studies have demonstrated that exposure to PM_2.5_ is associated with an increased risk of diabetes [9, 10], however, the underlying biological mechanisms have not been fully understood. Notably, recent studies showed that oral ingestion of inhalable particulate matter (PM_10_), ultrafine particulate matter (PM_0.1_), or chemicals present in ambient PM_2.5_ is sufficient to alter mouse gut microbiota [11–14] which could result in abnormal glucose metabolism. Although ingestion is not the primary route of exposure for ambient PM_2.5_ ambient particles that are inhaled and subsequently cleaned by the mucociliary escalator from the airway. Further studies are needed to determine whether inhalation exposure to ambient PM_2.5_ alters gut microbiota and subsequently causes abnormal glucose homeostasis. To this end, we exposed male C57Bl/6 J mice to concentrated ambient PM_2.5_ (CAP) or filtered air (FA) for 12 months, and analysed their glucose homeostasis and gut microbiota. The results demonstrate that chronic exposure to CAP significantly decreased the richness and composition of the faecal bacterial but not the fungal community. Furthermore, some of these alterations were significantly associated with abnormalities in glucose metabolism.

## Methods

### Animals and whole-body inhalation exposure to concentrated ambient PM_2.5_ (CAP)

3-week-old male C57Bl/6 J mice were purchased from the Animal Center of Shanghai Medical School, Fudan University (Shanghai, China). After 1-week acclimation, mice were subjected to exposure to FA (*n* = 10) or CAP (*n* = 10) using a versatile aerosol concentration enrichment system (VACES) that was modified for long-term whole-body exposures as previously described [15, 16]. The VACES was located on the campus of School of Public Health, Fudan University (130 Dong’an Road, Xuhui, Shanghai, China). The exposures were performed from March 2016 to March 2017. The exposure protocol comprised exposures for 8 h/day, 6 days/week (no exposure on Sunday). Ambient PM_2.5_ and CAP were collected weekly throughout the whole duration of exposure, and their elemental composition was determined by inductively coupled plasma mass spectroscopy (ICP-MS) for trace element analysis as previously described [17, 18]. During the period of exposure, mice were kept on a 12-h light/dark cycle at room temperature (20–25 °C) and 40–70% relative humidity, and received water and standard food ad lib. All procedures in the present study were approved by the institutional animal care and use committees of Fudan University, and all the animals were treated humanely and with regard for alleviation of suffering.

### Intraperitoneal glucose tolerance test (IPGTT)

After 46-week-exposure to FA/CAP, mice were subjected to IPGTT on that Sunday. Before testing, mice (50 weeks old) were fasted (initiated immediately after the Saturday’s exposure) for 16 h. On the day of experiments, the basal glucose level of tail vein blood was determined using an automatic glucometer (Glucotrend 2, Roche Diagnostics), and then mice were intraperitoneally injected with glucose (2 g/kg body weight). The glucose levels of the tail vein blood at 15, 30, 60, and 120 min after injection was measured as described above.

### Insulin tolerance test (ITT)

ITT was performed on mice after the 47-week-exposure to FA/CAP on that Sunday. Before testing, mice (51 weeks old) were fasted for 4 h. The basal glucose level of tail vein blood was determined using an automatic glucometer (Glucotrend 2, Roche Diagnostics) and then mice were intraperitoneally injected with insulin (0.5 U/kg body weight). The glucose levels of the tail vein blood at 15, 30, 60, and 120 min after injection was measured as described above.

### Faecal sample collection

After 48-week-exposure to FA/CAP, mice (52 weeks old) were immediately transferred to empty autoclaved metabolism cage (individually housed, no bedding), and allowed to defecate normally. The 24-h faecal pellets of each mouse were collected and stored in empty autoclaved 1.5 ml Eppendorf tubes using a sterile toothpick. The faecal samples were immediately placed on dry ice and then transferred to − 80 °C freezer. Samples were stored at − 80 °C until ready to extract DNA.

### DNA extraction and sequencing

The total genomic DNAs of the faecal samples were extracted using a MoBio PowerFecal DNA extraction kit (Qiagen) as per the manufacturer’s instructions. Briefly, the samples were homogenized in a 2 ml bead beating tube containing garnet beads. The lysis of host cells and microbial cells was facilitated by both mechanical collisions between beads and chemical disruption of cell membranes, ensuring efficient extraction from even the toughest microorganisms. The total genomic DNA was captured on a silica spin column and then eluted with 50 μl of elution buffer from the column after washing with washing buffer. These genomic DNA samples were stored at − 80 °C until the preparation of sequencing libraries. To prepare the sequencing libraries, the DNA concentration and quality was determined with a NanoDrop 1000 spectrophotometer (Thermo Scientific) and by agarose gel electrophoresis (1% wt/vol agarose in tris-acetate-EDTA buffer), respectively. One sample in FA group failed in this quality control (low DNA concentration), and therefore 9 FA and 10 CAP samples were subjected to library preparation and sequencing. The bacterial 16S rRNA gene V4 region and fungal ITS1 region were amplified by PCR using primers as follows: the bacterial community [19]: barcoded 515F (5′-GTG CCA GCM GCC GCG G-3′) and the reverse primer 907R (5’-CCG TCA ATT CM TTT RAG TTT-3′); the fungal community [20]: ITS1F (5’-CTT GGT CAT TTA GAG GAA GTA A-3′) and ITS2R (5’-GCT GCG TTC TTC ATC GAT GC-3′). Each 20 μl PCR reaction mix included 4 μl of 5× FastPfu buffer, 2 μl of 2.5 mM dNTPs, 0.8 μl of forward primer (5 μM), 0.8 μl of reverse primer (5 μM), 0.4 μl of FastPfu polymerase, 10 ng of template DNA, and ddH_2_O was added to make up the final volume to 20 μl. Thermal cycling was performed in a 9700 PCR System (ABI, GeneAmp 9700) with the following cycling: initial denaturation at 95 °C for 5 min followed by 27 cycles of denaturation at 95 °C for 30 s, annealing at 55 °C for 30 s, extension at 72 °C for 45 s, and a final extension at 72 °C for 10 min. All PCR products were subjected to agarose gel electrophoresis (2%) followed by purification using the AXYGEN gel extraction kit (Axygen). The purified amplicons were quantified using the Quant-iT PicoGreen dsDNA Assay Kit (Thermo Fisher) and QuantiFluor™-ST Blue-florescence quantitative system (Promega). They were then sequenced using the Illumina MiSeq system (Illumina) as per the manufacturer’s guidelines.

### Bioinformatics analysis

The primary sequencing data were saved in the Fastq format at SRA (Sequence Archive, http://www.ncbi.nlm.nih.gov/Traces/sra). All pyrosequencing reads were pre-processed based on the barcode and primer-end readers (PE readers) using Usearch software (version 7.1, http://drive5.com/uparse/). All reads recruited to the following analyses had the barcode, a minimal average quality score of 20, and maximally 2 mismatches within the primers. Additionally, all overlapped reads (a minimal overlap of 10 bp that had a mismatched rate of ≤0.2.) were merged. To avoid unnecessary computations, the repetitive sequences were extracted and discarded (http://drive5.com/usearch/manual/singletons.html). The optimized sequences were then clustered into operational taxonomics units (OTUs) using UCLUST followed by de novo OTU picking, and chimeras were removed using RDP gold database on Usearch software (version 7.1, http://drive5.com/uparse/). The bacterial and fungal taxonomy was assigned using Naïve Bayesian classifier in QIIME platform [21, 22] using SILVA database (Release 119, http://www.arb-silva.de) [23] and Unite fungal database (Release 6, http://unite.ut.ee/index.php) [24], respectively.

### Microbial diversity and richness analysis

OTU-based alpha diversity was estimated using four matrices of Mothur (www.mothur.org /wiki/Schloss_SOP#Alpha_diversity, version v.1.30): ACE, Chao-1, Shannon and Simpson. While ACE and Chao-1 estimators are used to reflect the total number of species in a sample, known as the richness of the community, Simpson and Shannon estimators are quantitative indicators of biodiversity in a region. The calculation of Simpson and Shannon estimators are based on different algorithms, and a larger Simpson estimator or a smaller Shannon estimator represents a lower community diversity. UniFrac distance analysis and principal co-ordinates analysis (Pcoa) using the relative abundance of OTUs were performed to estimate the beta diversity of community. In addition, linear discriminant effect size (LEfSe) analysis was used to find features differentially represented between the groups: a nonparametric factorial Kruskal-Wallis sum-rank test was used to detect significantly (*p* < 0.05) differential taxa, and the identified taxa were further subjected to a linear discriminant analysis (LDA) to evaluate the effect size of each single differential taxon.

### Mediation analysis

The mediation analysis was performed to evaluate the contribution of alteration in gut microbiota to CAP exposure-induced abnormal glucose metabolism. The total effect of CAP exposure on abnormality in glucose homeostasis (*X*) was assumed to be decomposed into a direct effect (*Y*) and an indirect effect (*M*) that is mediated by alteration in gut microbiota. [25] The mediation was then calculated based on two linear mixed effect (LME) models as demonstrated below [26]:

$$ {M}_i={\beta}_0+\alpha {X}_i+{\varepsilon}_i $$

$$ {Y}_i={\beta}_0^{\prime }+\lambda {M}_i+\theta {X}_i+{\eta}_i $$

Here *i* denotes subject (CAP exposure in the present study). *β*_0_ and ${\beta}_0^{\prime }$ are the intercepts for *M* and *Y*, respectively. The effect of *X* on *M* is designated as *α*, the effect of *M* on *Y* is designated as *λ*, and the direct effect of *X* on *Y* is designated as *θ*. *ε*_*i*_ and *η*_*i*_ are residuals for *M* and *Y*, respectively. The mediation analysis was conducted using R software (version 2.4.2, mediation package).

### Statistical analysis

All data were presented as mean ± SEM if not specified. Statistical significances were evaluated by student’s *t* test or ANOVA analysis (with Bonferroni post-test) using GraphPad Prism Software (version 5), and a *p* < 0.05 was set as a significance. The area under curve (AUC) for each mouse’s IPGTT and ITT data were calculated using GraphPad Prism Software (version 5. Y_baseline_ = 0, all peaks must go above the baseline, and ignore peaks that are less than 10% of the distance from minimum to maximum Y). The Spearman rank correlation and the Pearson correlation analyses were performed using GraphPad Prism Software (version 5).

## Results

### Chronic exposure to CAP results in glucose and insulin intolerance

**Fig. 1:**
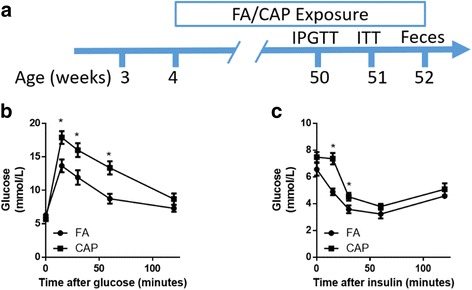
Chronic exposure to CAP results in glucose intolerance and insulin resistance. **a** The experimental scheme. **b** the response curves of IPGTT after 12-month-exposure to FA/CAP. *p < 0.05 versus FA at the same time point, repeated measures two-way ANOVA. **c** the response curves of ITT after 12-month-exposure to FA/CAP. *p < 0.05 versus FA at the same time point, repeated measures two-way ANOVA

Figure 1a depicts the experimental scheme. The average concentration of ambient PM_2.5_ during this period was 42.1 ± 23.5 μg/m^3^, and the average PM_2.5_ concentrations in FA and CAP chambers were 12.3 ± 5.8 and 276.2 ± 170.1 μg/m^3^, respectively. As the exposure was performed for 8 h/day and 6 days/week, the 24-h average PM_2.5_exposure levels for FA- and CAP-exposed mice during this period were 33.6 and 109.0 μg/m^3^, respectively. The exposure level of the CAP group was markedly higher than the national ambient air quality standards of China (35 μg/m^3^), but was common in areas with heavy air pollution such as Beijing, China [27]. Table 1 shows that the elemental composition of PM_2.5_ in the CAP chamber was comparable to that of ambient PM_2.5_, suggesting the technique efficiently concentrated the PM without altering its composision. The relatively high crustal elements including Si, Al, Ti, and Fe in PM_2.5_ [28] reflected the undergoing major construction on this campus.

**Table 1:** Ambient PM_2.5_ and CAP samples were collected weekly, and the elemental composition was determined by ICP-MS

	Ambient		CAP	
	μg/m^3^	%	μg/m^3^	%
Na	0.12 ± 0.02	0.29	1.06 ± 0.41	0.38
Mg	0.94 ± 0.05	2.23	5.65 ± 0.83	2.05
Al	3.09 ± 0.13	7.33	18.83 ± 2.25	6.82
Si	11.51 ± 0.47	27.32	71.42 ± 6.9	25.86
P	0.04 ± 0.01	0.09	0.33 ± 0.09	0.12
S	0.44 ± 0.27	1.05	6.41 ± 5.84	2.32
Cl	0.02 ± 0.03	0.04	0.74 ± 1.01	0.27
K	0.41 ± 0.06	0.97	3.16 ± 0.84	1.14
Ca	21.62 ± 0.64	51.32	139.19 ± 5.96	50.40
Ti	0.94 ± 0.02	2.23	6.28 ± 0.11	2.27
Cr	0.02 ± 0.003	0.06	0.17 ± 0.03	0.06
Mn	0.01 ± 0.01	0.02	0.21 ± 0.29	0.08
Fe	1.5 ± 0.04	3.59	11.7 ± 2.61	4.24
Ni	0.01 ± 0.003	0.03	0.11 ± 0.06	0.04
Cu	0.01 ± 0.002	0.01	0.07 ± 0.06	0.03
Zn	0.06 ± 0.01	0.13	0.96 ± 0.79	0.35
Ga	0.02 ± 0.001	0.04	0.13 ± 0.01	0.05
As	0.24 ± 0.01	0.57	1.63 ± 0.05	0.59
Br	0.003 ± 0.005	0.01	0.18 ± 0.24	0.06
Rb	0.01 ± 0.001	0.02	0.07 ± 0.01	0.02
Sr	0.37 ± 0.02	0.89	2.44 ± 0.07	0.88
Y	0.01 ± 0.001	0.02	0.06 ± 0.01	0.02
Zr	0.2 ± 0.01	0.47	1.4 ± 0.05	0.51
Nb	0.01 ± 0.001	0.02	0.05 ± 0.001	0.02
Sn	0.005 ± 0.004	0.00	0.01 ± 0.01	0.00
Sb	0.005 ± 0.006	0.01	0.04 ± 0.02	0.01
Ce	0.21 ± 0.02	0.51	1.49 ± 0.03	0.54
Pr	0.03 ± 0.01	0.07	0.22 ± 0.02	0.08
Eu	0.05 ± 0.01	0.13	0.37 ± 0.06	0.14
Gd	0.01 ± 0.01	0.02	0.04 ± 0.03	0.01
Tb	0.08 ± 0.01	0.19	0.38 ± 0.18	0.14
Er	0.01 ± 0.005	0.03	0.17 ± 0.15	0.06
Lu	0.02 ± 0.003	0.05	0.23 ± 0.13	0.08
W	0.004 ± 0.003	0.01	0.03 ± 0.02	0.01
Ir	0.002 ± 0.002	0.00	0.002 ± 0.001	0.00
Hg	0.01 ± 0.001	0.02	0.05 ± 0.01	0.02
Pb	0.01 ± 0.006	0.03	0.15 ± 0.1	0.05
Bi	0.002 ± 0.001	0.01	0.03 ± 0.01	0.01
U	0.08 ± 0.02	0.18	0.73 ± 0.16	0.27

Chronic exposure to CAP has been shown to result in glucose intolerance and insulin resistance in various mouse models [29, 30]. Figure 1b and c reveal that consistent with these previous studies, the 12-month exposure to CAP versus FA significantly impaired mouse glucose tolerance and induced a marked insulin resistance.

### Chronic exposure to CAP alters the richness of gut bacterial community

Increasing evidence has indicated that the gut microbiota plays a crucial role in the homeostatic regulation of glucose metabolism [31]. To investigate whether exposure to ambient PM_2.5_ causes gut dysbiosis and subsequently abnormal glucose metabolism, we collected faecal samples from the FA- or CAP-exposed mice, and characterized their bacterial and fungal communities through Illumina amplicon sequencing. The bacterial genomic DNA sequencing obtained 752,834 raw reads in total. After merging overlaps and removing repetitive reads, an average of 36,600 high quality sequences was identified and clustered into 511 OTUs. The fungal genomic DNA sequencing generated 921,548 raw reads in total. An average of 38,153 high quality sequences was identified and clustered into 669 OTUs.

**Fig. 2:**
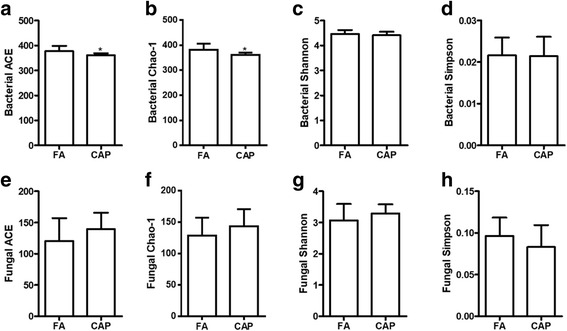
Chronic exposure to CAP alters the richness but not diversity of faecal bacterial community. Male C57Bl/6 J mice were exposed to FA or CAP for 12 months and faecal samples were collected and subjected to microbiomics analysis. **a-d** the alpha diversity estimators of faecal bacterial community. **e-h** the alpha diversity estimators of faecal fungal community. *n* = 9 or 10/group. *p < 0.05 versus FA, student *t* test

The community richness and diversity estimators represent the integral level of dysbiosis of gut microbiota and predict the development of abnormal glucose metabolism [6]. Figure 2a and b demonstrate that chronic exposure to CAP significantly reduced the faecal bacterial ACE and Chao-1 estimators (the most frequently used indexes of community richness). In contrast, CAP exposure did not significantly influence the Shannon and Simpson estimators of faecal bacterial community (the indexes of the community diversity, Fig. 2c and d). Figure 2e-h show that CAP versus FA exposure did not significantly alter any α richness and diversity estimator of the faecal fungal community.

### Chronic exposure to CAP alters the composition of gut bacterial and fungal communities

**Fig. 3:**
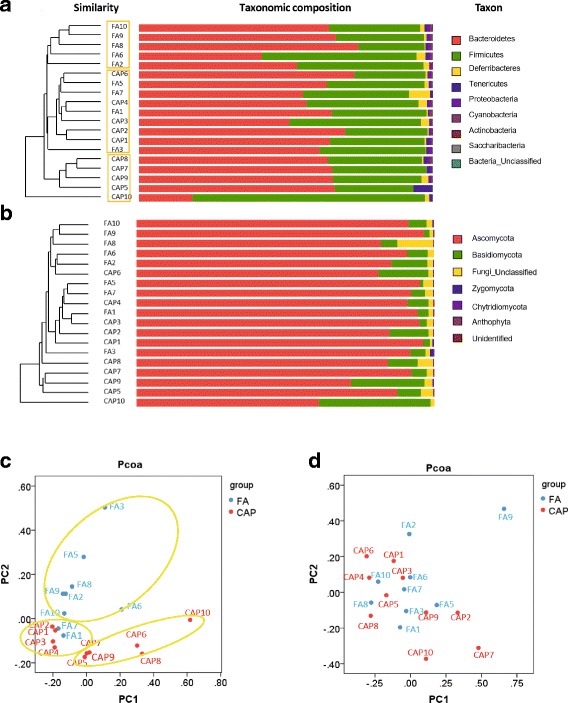
Chronic exposure to CAP alters the composition of faecal bacterial community. **a** and **b** the phyla composition of mouse faecal microbiota after 12 months exposure to FA or CAP. Hierarchical clustering analysis was conducted using taxonomical data. **c** and **d** the Pcoa using unweight UniFrac values

To further document the effects of CAP exposure on the gut bacterial and fungal communities, hierarchical clustering analyses using taxonomical data were conducted. Figure 3a reveals three clusters by the hierarchical clustering of the bacterial community composition: one is composed of about half FA-exposed samples (FA2, FA3, FA6, FA8, FA9 and FA10); another contains about half CAP-exposed samples (CAP5, CAP7, CAP8, CAP9 and CAP10); and the third includes all the remaining samples (FA1, FA5, FA7, CAP1, CAP2, CAP3, CAP4 and CAP6). These results suggested that in spite of the marked individual variation, the clustering of samples was evident. This clustering was corroborated by principal coordinate analysis (Pcoa) using unweighted UniFrac values (Fig. 3c). In contrast, no evident clustering of samples was observed when performing hierarchical clustering and Pcoa using the fungal community composition data (Fig. 3b and d).

Figure 3a shows that the gut bacterial community of the FA- or CAP-exposed mice was primarily comprised of Bacteroidetes (60.4 ± 12.9%), Firmicutes (36.0 ± 12.7%), Deferribacteres (1.5 ± 1.7%), Tenericutes (0.9 ± 1.3%), Proteobacteria (0.7 ± 0.3%), and Cyanobacteria (0.3 ± 0.3%). The gut fungal community (Fig. 3b) comprised primarily Ascomycota (87.9 ± 8.9%), Basidiomycota (9.2 ± 9.0%), unclassified (2.7 ± 2.6%), and Zygomycota (0.2 ± 0.3%). CAP versus FA exposure did not result in any significant difference in the relative abundance of these bacterial and fungal phyla.

**Fig. 4:**
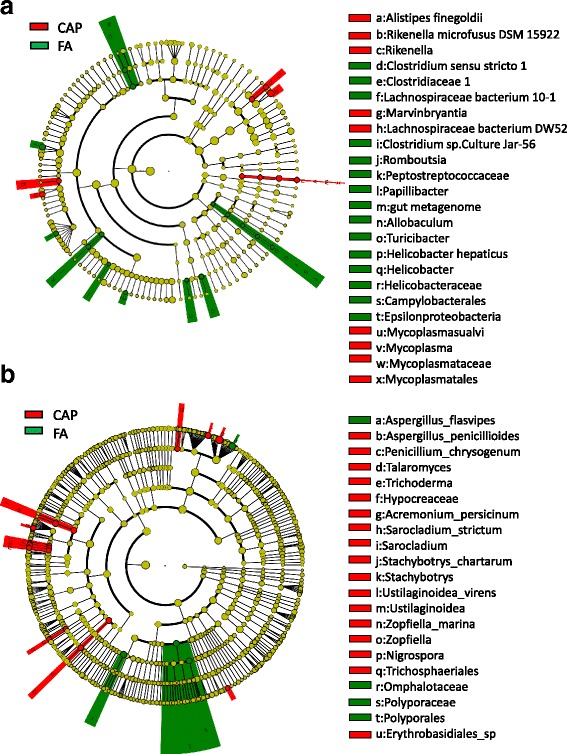
The differential taxa between FA- and CAP-exposed mice. **a** the cladogram of faecal bacterial community. **b** the cladogram of faecal fungal community

To document the impact of CAP exposure on the composition of gut microbiota, we performed LEfSe analyses using the relative abundance data to identify the bacterial and fungal taxa differentially represented between FA- and CAP-exposed groups. Figure 4 shows that there were 24 differential bacterial taxa and 21 differential fungal taxa. Specifically, CAP exposure significantly increased the relative abundance of 9 bacterial and 17 fungal taxa, and significantly decreased the relative abundance of 15 bacterial and 4 fungal taxa.

### The association between CAP exposure-induced alterations in gut microbiota and abnormalities in glucose metabolism

**Fig. 5:**
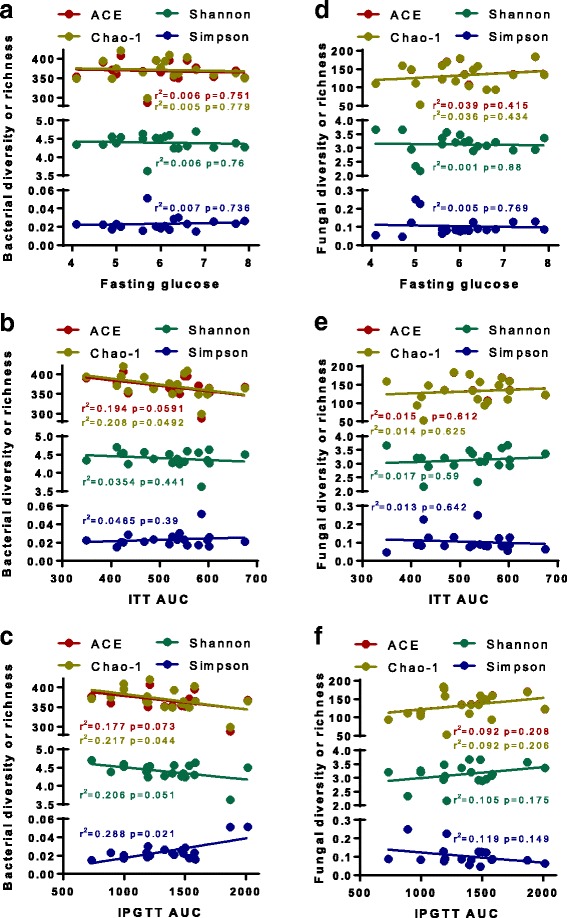
The correlations between the richness and diversity of faecal microbiota and the indictors of glucose homeostasis. The Pearson’s correlation analyses were performed between the fasting glucose level (**a** and **d**), the area under curve (AUC) of ITT (**b** and **e**), the AUC of IPGTT (**c** and **f**), and the alpha diversity estimators of faecal bacterial (**a-c**) or fungal (**d-f**)

To test whether the alteration in gut microbiota mediates CAP exposure-induced abnormalities in glucose metabolism, we performed correlation analyses using the richness and diversity estimators of gut bacterial community and their indicators of glucose homeostasis. Figure 5a shows that while there was no significant correlation between the α diversity estimators of gut bacterial community and the fasting glucose level, the ACE and Chao-1 estimators of gut bacterial community appeared to be associated with insulin sensitivity (Fig. 5b), and all four α diversity estimators appeared to be correlated with glucose tolerance (Fig. 5c). In contrast, we did not observe any significant correlation between the richness and diversity estimators of gut fungal community and their indicators of glucose homeostasis (Fig. 5d-f).

To further document whether CAP exposure-induced abnormalities in glucose homeostasis can be attributable to change in any single bacterial or fungal taxon, we performed Spearman rank correlation analyses using the indicators of glucose homeostasis and the relative abundances of differential bacterial and fungal taxa. Table 2 reveals that there was no significant correlation between the relative abundance of differential bacterial taxa and the fasting glucose level, but the relative abundances of 14 and 20 differential bacterial taxa were significantly correlated with IPGTT AUC and ITT AUC, respectively. Of these, 2 and 9 bacterial taxa were positively correlated with IPGTT AUC and ITT AUC, respectively. In contrast, there were only 5 significant correlations between the relative abundance of differential fungal taxa and the indicators of mouse glucose homeostasis (Table 3).

**Table 2:** The bacterial taxa significantly different between FA- and CAP-exposed mice (LEfSe analysis) and significantly correlated with the indicator of glucose metabolism (Spearman correlation analysis). *n* = 9 or 10/group

	Rank	Taxon	LEfSe			Spearman	
			mean(FA)	mean(CAP)	*p*-value	rho	*p*-value
IPGTT AUC	Class	Epsilonproteobacteria	0.00012	0.00000	0.013	−0.537	0.018
	Order	Campylobacterales	0.00012	0.00000	0.013	− 0.537	0.018
	Family	Helicobacteraceae	0.00012	0.00000	0.013	−0.537	0.018
		Peptostreptococcaceae	0.00041	0.00000	0.013	−0.591	0.008
	Genus	Clostridium sensu stricto 1	0.00088	0.00000	0.013	−0.540	0.017
		Helicobacter	0.00012	0.00000	0.013	−0.537	0.018
		Romboutsia	0.00041	0.00000	0.013	−0.591	0.008
		Turicibacter	0.00056	0.00000	0.013	−0.551	0.015
	Species	Clostridium sensu stricto 1_uncultured bacterium	0.00088	0.00000	0.013	−0.540	0.017
		Helicobacter hepaticus	0.00012	0.00000	0.013	−0.537	0.018
		Romboutsia_uncultured bacterium	0.00041	0.00000	0.013	−0.591	0.008
		Ruminiclostridium 5_Unclassified	0.00075	0.00191	0.017	0.465	0.045
		Ruminiclostridium 5_uncultured Clostridiales bacterium	0.00017	0.00037	0.010	0.458	0.049
		Turicibacter_uncultured bacterium	0.00056	0.00000	0.013	−0.551	0.015
ITT AUC	Class	Epsilonproteobacteria	0.00012	0.00000	0.013	− 0.468	0.043
	Order	Campylobacterales	0.00012	0.00000	0.013	−0.468	0.043
		Mycoplasmatales	0.00000	0.00338	0.028	0.487	0.035
	Family	Helicobacteraceae	0.00012	0.00000	0.013	−0.468	0.043
		Mycoplasmataceae	0.00000	0.00338	0.028	0.487	0.035
		Peptostreptococcaceae	0.00041	0.00000	0.013	−0.610	0.006
	Genus	Clostridium sensu stricto 1	0.00088	0.00000	0.013	−0.572	0.011
		Helicobacter	0.00012	0.00000	0.013	−0.468	0.043
		Mycoplasma	0.00000	0.00338	0.028	0.487	0.035
		Romboutsia	0.00041	0.00000	0.013	−0.610	0.006
		Turicibacter	0.00056	0.00000	0.013	−0.559	0.013
	Species	Alistipes finegoldii	0.00912	0.03625	0.013	0.692	0.001
		Clostridium sensu stricto 1_uncultured bacterium	0.00088	0.00000	0.013	−0.572	0.011
		Helicobacter hepaticus	0.00012	0.00000	0.013	−0.468	0.043
		Marvinbryantia_uncultured bacterium	0.00002	0.00050	0.035	0.539	0.017
		Mycoplasma sualvi	0.00000	0.00338	0.028	0.487	0.035
		Rikenella microfusus DSM 15922	0.00000	0.00067	0.028	0.496	0.031
		Romboutsia_uncultured bacterium	0.00041	0.00000	0.013	−0.610	0.006
		Ruminiclostridium 5_Unclassified	0.00075	0.00191	0.017	0.610	0.006
		Turicibacter_uncultured bacterium	0.00056	0.00000	0.013	−0.559	0.013

**Table 3:** The fungal taxa significantly different between FA- and CAP-exposed mice (LEfSe analysis) and significantly correlated with the indicator of glucose metabolism (Spearman correlation analysis). *n* = 9 or 10/group

	Rank	Taxon	LEfSe			Spearman	
			mean(FA)	mean(CAP)	*p*-value	rho	*p*-value
Fasting glucose	Species	Aspergillus_flavipes	0.03071	0.00118	0.017	0.578	0.010
IPGTT AUC	Species	Aspergillus_penicillioides	0.12700	0.23508	0.001	0.493	0.032
ITT AUC	Genus	Talaromyces	0.00457	0.00840	0.022	0.526	0.021
	Species	Aspergillus_penicillioides	0.12700	0.23508	0.001	0.667	0.002
		Talaromyces_Unclassified	0.00447	0.00816	0.017	0.511	0.026

To statistically assess how much CAP exposure-induced abnormalities in glucose homeostasis is accounted for by changes in the gut microbiota, we performed the mediation analyses. Table 4 shows that the change in the Chao-1 estimator of the gut bacterial community accounted for 15% of CAP exposure-induced change in IPGTT AUC. No other significant mediation was observed.

**Table 4:** The mediation analysis. *n* = 9 or 10/group

	Glucose metabolism	ACE		Chao-1		Shannon		Simpson	
	indicator	Proportion mediated	*p*-value	Proportion mediated	*p*-value	Proportion mediated	*p*-value	Proportion mediated	*p*-value
Bacteria	Fasting glucose	−0.118	0.56	−0.127	0.44	−0.092	0.68	−0.024	0.92
	IPGTT AUC	0.065	0.56	0.154	0.04	0.115	0.56	0.052	0.48
	ITT AUC	0.032	0.88	0.058	0.72	0.023	0.88	0.013	0.72
Fungus	Fasting glucose	−0.29	0.28	−0.216	0.36	−0.104	0.56	−0.224	0.44
	IPGTT AUC	0.022	0.72	0.036	0.76	0.013	0.96	0.1	0.4
	ITT AUC	−0.033	0.76	−0.017	0.72	−0.078	0.64	−0.04	0.8

## Discussion

Exposure to ambient PM_2.5_ correlates to increased insulin resistance and/or impaired glucose tolerance and thus is implicated in the pathogenesis of diabetes [10]. Emerging evidence has demonstrated that the gut microbiota may play an important role in the maintenance of glucose homeostasis [31]. However, it remains to be determined whether PM_2.5_ exposure impacts the gut microbiota and if its adverse health effects are mediated through this alteration. In the present study, we demonstrated that chronic exposure to CAP induced marked insulin resistance and impaired glucose tolerance in C57Bl/6 J mice in association with a decrease in the richness in (ACE and Chao-1) estimators of faecal bacterial but not fungal communities, and alteration in the composition of gut microbiota. As such, the present data highlight a role of the gut microbiota in the development of adverse health effects due to exposure to ambient PM_2.5_, particularly abnormalities in glucose homeostasis.

The role of gut microbiota in the development of a multitude of complex human diseases is attracting attention in many relevant scientific communities [31]. However, while exposure to ambient PM_2.5_ has been shown to correlate with the majority of these diseases, its effect on the gut microbiota remains unclear. To the best of our knowledge, this is the first study showing significant effects of inhalation exposure to CAP on the gut microbiota. Oral ingestion of inhalable particulate matter (PM_10_), ultrafine particulate matter (PM_0.1_), or chemicals present in ambient PM_2.5_ has been shown to alter mouse gut microbiota [12–14]. Although oral ingestion is not the primary route of exposure for ambient PM_2.5_ pollution, PM_2.5_ deposited in the lower airway may be cleared through the combined action of phagocytic cells (macrophages and granulocytes and the mucociliary escalator, with a marked proportion of PM_2.5_ ultimately being ingested. Therefore, these studies [12–14] not only are consistent with the present data but also provide potential mechanisms for the impact of PM_2.5_ inhalation on the gut microbiota. However, caution should be taken when interpreting these data as whether PM_2.5_ inhalation impacts the gut microbiota through the ingested PM_2.5_ remains to be determined.

The richness and diversity estimators represent the integral status of gut microbiota and appeared to be better predictors for abnormal glucose metabolism than the relative abundance of single taxon [6]. While the richness reflects the number of species per sample (the more species present in a sample, the ‘richer’ the sample), the diversity depends not only on richness, but also on the relative abundance of the different species making up the richness of a sample. In the present study, we showed that exposure to CAP significantly decreased the richness estimators of gut bacterial community (Fig. 2a and b), which is consistent with the absence of many bacterial taxa in the gut microbiota of CAP-exposed animals (Table 2). In contrast, we did not observe any significant effect of CAP exposure on the bacterial diversity as indicated by the Shannon and Simpson estimators. This is consistent with the present data showing no significant difference in the relative abundances of bacterial phyla (Fig. 3). The lack of effect on the diversity of gut bacterial community is in direct contrast to the marked effects of ingestion of PM_10_ or PM_0.1_ on the relative abundance of phyla [12, 13]. This inconsistency may reflect the difference in the routes of exposure, re-emphasizing serious consideration of the route of exposure in PM_2.5_ toxicological studies.

In the present study, we corroborated the glucose intolerance and insulin resistance following chronic exposure to CAP (Fig. 1), reaffirming the glucose-metabolic effect of PM_2.5_ exposure [32]. Importantly, the present study revealed an association between CAP-induced glucose intolerance and alteration in the bacterial richness (Table 4), suggesting that the gut microbiota plays a role in the development of diabetes due to exposure to ambient PM_2.5_. In contrast, we did not observe any significant mediation of abnormalities in glucose homeostasis by alteration of single bacterial taxon. This is in accordance with the above-mentioned notation that the richness and diversity estimators may be better predictors for abnormal glucose metabolism than the abundance of single taxon [6].

In addition to the alteration in richness of gut microbiota, the present study also calls attention to the role of some specific species in CAP exposure-induced abnormal glucose metabolism. For example, we showed that Clostridium sensu strito 1 was absent in CAP-exposed mice and meanwhile negatively correlated with mouse glucose intolerance and insulin resistance, strongly supporting its implication in CAP exposure-induced diabetes. This is perfectly consistent with its anti-diabetic effects in humans and animal models [33]. The present study also showed that Helicobacter hepaticus was absent in CAP-exposed mice and negatively correlated with mouse glucose intolerance and insulin resistance. However, given that it is positively correlated with Vitamin D deficiency-induced glucose intolerance [34], further study is needed to verify its role in CAP exposure-induced insulin resistance and glucose intolerance. Moreover, the present study revealed that in addition to the two species above, several other gut microbe species were significantly altered by CAP exposure and correlated with glucose intolerance and/or insulin resistance (Table 2). However, their role in the host’s glucose homeostasis regulation has not yet been investigated, warranting further studies to examine these correlations.

Compared to the gut bacterial community, the gut fungal community draws less attention in the diabetes area. Interestingly, the present study showed comparable numbers of bacterial and fungal differential taxa (Fig. 4). It is however noteworthy that CAP exposure decreased the abundance of most differential bacterial taxa but increased the abundance of most differential fungal taxa. Given the various examples of antagonism between bacteria and fungi [35], these apparently opposite effects of CAP exposure may represent another antagonism between bacteria and fungi and thus warrant further studies to determine which the primary effect of CAP exposure is. In addition, mediation analyses showed that the decrease in the richness of gut bacterial community partly accounted for CAP exposure-induced impairment of glucose tolerance, and correlation analyses revealed much more bacterial versus fungal differential taxa that were significantly correlated with the indicators of mouse glucose metabolism (Tables 3 and 4).

Although the present study demonstrates a marked impact of CAP exposure on the gut microbiota and implicates it in the development of diabetes due to exposure to ambient PM_2.5_, several limitations should be noted. Firstly, we did not investigate the causality between CAP exposure-induced dysbiosis and abnormalities in glucose homeostasis. This will require using antibiotics-treated and/or germ-free mice. Secondly, we did not ascertain the time- and dose-dependency of this CAP exposure-induced dysdiosis, which are valuable for the delineation of role of gut microbiota in the development of adverse health effects due to exposure to PM_2.5_. Thirdly, while ingestion of ambient particles was shown to alter the composition and function of gut microbiota [11–14], how PM_2.5_ inhalation may impact the gut microbiota and subsequently contribute to abnormalities in glucose metabolism remains to be determined. Inflammation is widely believed to be central in the development of adverse health effects due to exposure to PM_2.5_ [36]. Notably, inflammatory diseases such as multiple sclerosis and arthritis have been shown to be correlated with changes in the gut microbiota [37], suggesting that inflammation may play a role in the alteration of gut microbiota due to exposure to PM_2.5_. Also, given the different feeding and pathologic patterns between humans and mice, human studies are needed to confirm the specialized role of gut microbiota in the development of abnormal glucose metabolism due to exposure to ambient PM_2.5_.

## Conclusions

In the present study, we demonstrated that inhalation exposure to PM_2.5_ induced abnormal glucose homeostasis and change in the composition of gut microbiota. Their strong correlation suggests that the gut microbiota may be crucial for PM_2.5_ exposure-induced metabolic disorders. As such, our results suggest a novel mechanism for PM_2.5_ exposure-induced adverse health effects and thus provide potential targets for the development of effective prevention and/or treatment.

## Abbreviations

AUC: Area under curve
BCAA: Branched-chain amino acid
CAP: Concentrated ambient PM_2.5_
FA: Filtered air
ICP-MS: Inductively coupled plasma mass spectroscopy
IPGTT: Intraperitoneal glucose tolerance test
ITT: Insulin tolerance test
LDA: Linear discriminant analysis
LEfSe: Linear discriminant effect size
LME: Linear mixed effect
OTUs: Operational taxonomics units
Pcoa: Principal co-ordinates analysis
PM_0.1_: Ultrafine particulate matter
PM_10_: Inhalable particulate matter
PM_2.5_: Ambient fine particulate matter
T2D: Type 2 diabetes
VACES: Versatile aerosol concentration enrichment system
